# Manipulating Amino Acid Metabolism to Improve Crop Nitrogen Use Efficiency for a Sustainable Agriculture

**DOI:** 10.3389/fpls.2020.602548

**Published:** 2020-11-30

**Authors:** Younès Dellero

**Affiliations:** IGEPP, INRAE, Institut Agro, Univ Rennes, Le Rheu, France

**Keywords:** source-sink relationships, senescence, amino acid, catabolism, assimilation, transport, nitrogen use efficiency (NUE), crop plant

## Abstract

In a context of a growing worldwide food demand coupled to the need to develop a sustainable agriculture, it is crucial to improve crop nitrogen use efficiency (NUE) while reducing field N inputs. Classical genetic approaches based on natural allelic variations existing within crops have led to the discovery of quantitative trait loci controlling NUE under low nitrogen conditions; however, the identification of candidate genes from mapping studies is still challenging. Amino acid metabolism is the cornerstone of plant N management, which involves N uptake, assimilation, and remobilization efficiencies, and it is finely regulated during acclimation to low N conditions and other abiotic stresses. Over the last two decades, biotechnological engineering of amino acid metabolism has led to promising results for the improvement of crop NUE, and more recently under low N conditions. This review summarizes current work carried out in crops and provides perspectives on the identification of new candidate genes and future strategies for crop improvement.

## Introduction

More than half of the world’s population are fed by crops grown with the addition of synthetic nitrogen (N) fertilizers (Zhang et al., 2015). This supply of N coupled to other essential nutrients has allowed farmers to increase crop production per unit to meet food demand over the last century. However, nearly 50% of N fertilizers applied to the field are not used by crops, independently of world region or crop type. This N surplus has led to disastrous consequences that threaten the sustainability of agricultural production systems. The application of inorganic N fertilizers in agricultural areas has largely contributed to the increase of soil acidification, gaseous ammonia emissions in the atmosphere, and nitrate levels in our water resources, with dangerous consequences for human health (Behera et al., 2013; Ward et al., 2018). Therefore, it is crucial to improve crop N use efficiency (NUE) while reducing N inputs to the field to meet the growing food demand in a sustainable agriculture context. Plant NUE can be defined as the maximal quantity of seeds or biomass (depending on downstream applications) obtained from a defined amount of N supplied to plants (Xu et al., 2012). Plant NUE comprises the capacity to take up N from the soil (N uptake efficiency, NUpE) and the capacity to utilize this N efficiently within the plant to produce the harvested product (N utilization efficiency, NUtE). NUtE integrates the capacity to assimilate inorganic N into carbon (C) skeletons (N assimilation efficiency, NAE) to produce amino acids (AA) and subsequently essential N-containing molecules (proteins, DNA, RNA, chlorophylls, etc.) and the capacity to remobilize assimilated N from source-to-sink tissues (N remobilization efficiency, NRE; Avice and Etienne, 2014). AA metabolism is the cornerstone of plant N management since it is actively involved in both NUpE and NUtE through AA and protein biosynthesis, senescence-induced degradation of proteins into AAs, and overall source-to-sink AA transport (Tegeder and Masclaux-Daubresse, 2018). Comparison of pool sizes between free AAs and AAs in proteins nicely highlights the fine regulation exerted on AA metabolism to support primary metabolism and plant growth (Hildebrandt et al., 2015). Regulation of AA metabolism also plays an active role during acclimation to low N conditions and other abiotic stresses including heat, cold, dark, drought, and salt (Kant et al., 2011; Hildebrandt, 2018). Therefore, AA metabolism is an interesting target for the improvement of crop NUE in both sustainable agriculture and future climate change contexts. The use of classical genetic approaches based on natural allelic variations existing within plant species and varieties for this purpose have already been undertaken for rice, maize, wheat, barley, and rapeseed (Han et al., 2015; Bouchet et al., 2016). While some quantitative trait loci (QTL) associated with crop NUE under low N conditions have been found, the identification of determinant candidate genes from mapping studies is still a challenge. Over the two last decades, genetic engineering of AA metabolism has given some promising results with respect to the improvement of crop NUE and more recently under low N conditions. This review summarizes current and promising future targets for the genetic manipulation of AA metabolism in crops with respect to NUE improvement and tolerance to low N conditions and other abiotic stresses.

## Ammonia Assimilation

Nitrogen is first taken up from the soil by plant roots in the form of nitrate ions, ammonium ions, and AAs. Prior to be assimilated, nitrate is reduced to nitrite in the cytosol, which is then reduced to ammonium in plastids. This reduction can occur either directly in the roots or in the leaves after a transport step *via* the xylem (Masclaux-Daubresse et al., 2010; Yoneyama and Suzuki, 2019). In plants, the major ammonia assimilation route is *via* the glutamine synthetase/glutamate:2-oxoglutarate aminotransferase (GS/GOGAT) cycle; however, asparagine synthetase (ASN) and glutamate dehydrogenase (GDH) can also participate depending on plant status (Masclaux-Daubresse et al., 2010).

### Glutamine Synthetase/Glutamate:2-Oxoglutarate Aminotransferase Cycle

GS catalyzes the ATP-dependent condensation of ammonia and Glu to form Gln. Then, GOGAT transfers the amide group of Gln to 2-oxoglutarate to produce two molecules of Glu with the consumption of reducing power (either as reduced ferredoxin or as NADH). There are two types of GS enzyme in plants: GS1 and GS2. In *Arabidopsis*, a single gene (*GLN2*) encodes the chloroplast-localized GS2 isoform that is mainly responsible for ammonia assimilation in photosynthetic tissues and for the reassimilation of photorespiratory ammonia released by the activity of mitochondrial glycine decarboxylase complex (GDC; Wallsgrove et al., 1987). In *Arabidopsis*, GS1 is encoded by five genes (*GLN1-1* to *GLN-5*). While GS1 enzymes are located to the cytosol, their expression patterns in leaves, roots, and phloem companion cells can significantly vary depending on environmental conditions. GS1 enzymes play a key role in roots for ammonium assimilation and in vascular tissues for N reallocation as Gln to sink tissues during senescence (Martin et al., 2006; Lothier et al., 2011; Avila-Ospina et al., 2015). There are also two types of GOGAT in plants: Ferredoxin-GOGAT (Fd-GOGAT), present in leaf chloroplasts and encoded by two genes in *Arabidopsis* (*GLU1* and *GLU2*) and NADH-GOGAT, present in plastids of phloem companion cells and encoded by a single gene in *Arabidopsis* (*GLT1*; Suzuki and Knaff, 2005).

**Figure 1:**
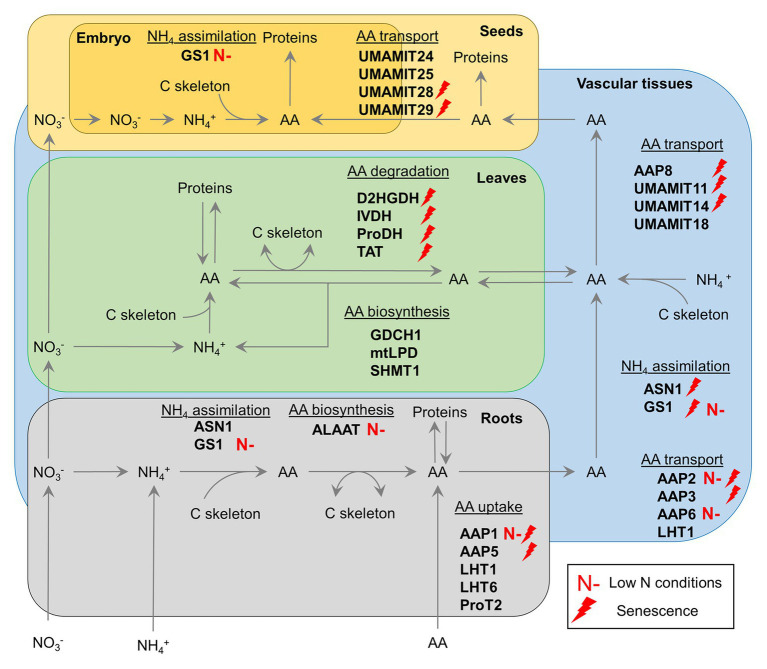
Current and promising targets for genetic manipulation of amino acid metabolism and transport to improve crop nitrogen use efficiency for a sustainable agriculture. Red lightning refers to gene targets known to be upregulated during senescence. Red “N-” refers to targets with a known biotechnological potential in low N conditions in certain crops. AAP, amino acid permease; ALAAT, alanine:2-oxoglutarate aminotransferase; ASN, asparagine synthetase; D2HGDH, D-2-hydroxyglutarate dehydrogenase; GDCH, H-protein of the glycine decarboxylase complex; GS, glutamine synthetase; IVDH, isovaleryl-CoA dehydrogenase; LHT, lysine histidine transporter; mtLPD, mitochondrial L-protein of the glycine decarboxylase complex; ProDH, proline dehydrogenase; ProT, proline transporter; SHMT, serine hydroxymethyl aminotransferase; TAT, tyrosine aminotransferase; UMAMIT, usually multiple amino acid move in and out transporter.

GS1 has attracted attention for many years as a potential target for the improvement of crop NUE, due to its central role in ammonia assimilation in roots and N remobilization during senescence (Figure 1; Thomsen et al., 2014). Indeed, this last role has been reinforced recently by the characterization of a triple *gln1-1/gln1-2/gln1-3* mutant of *Arabidopsis*. This multiple T-DNA insertion line had a lower seed yield and N remobilization activity from cauline and rosette leaves to seeds under both high and low N conditions (Moison et al., 2018).

In terms of biotechnological applications, a major strategy was to constitutively overexpress specific *GS1* cDNAs under the control of a *CaMV35S* promoter to improve NUpE and NAE in crops under low N conditions (Table 1). From a global point of view, some interesting results were obtained for *Lotus japonicus*, *Nicotiana tabacum*, and *Zea mays* in terms of NUE. However, significant differences were found depending on the *GS1* genes used. Indeed, overexpression of *GS1-5* from *Glycine max* in *Nicotiana tabacum* decreased plant growth and pod number per plant while only the overexpression of *GS1* genes from *Pisum sativum* succeeded to increase plant biomass under high, moderate, and low N conditions. Since GS1 activity seemed to be rate-limiting for NAE in *Nicotiana tabacum*, a possible explanation for these differences was that the efficient regulation of the assembly/disassembly of GS1 subunits by phosphorylation, nitration and the redox state of the cells may depend on specific exogenous GS1 isoforms (Thomsen et al., 2014). That said, toxicity of a strong GS1 activity in multiple organs has been observed for *Pisum sativum* and *Oryza sativa*. Indeed, GS1 activity has a high energetic cost and can stimulate the import of ammonium from roots thereby contributing to its toxic accumulation. Nevertheless, the simultaneous overexpression of *OsGS1.1* and *OsGS2* in *Oryza sativa* increased NAE and plant biomass (Table 1). This surprising result suggested that the GS2 could be a better candidate than GS1 isoforms for NUE improvement. To address the negative effects of GS1 overexpression, either organ-specific or moderate promoters have been used. These strategies were successful for *Sorghum bicolor* and especially *Pisum sativum*, where the root-specific expression of *GS1-5* from *Glycine max* increased plant biomass and N uptake in both high and low N conditions. Recently, the impact of GS1 overexpression has been investigated in *Nicotiana tabacum*, *Oryza sativa*, and *Hordeum vulgare* during abiotic stresses such as drought, salt stress, osmotic stress or elevated CO_2_ combined with N limitations. The results indicated that GS1 was a suitable target for improving NUE, grain yield, and tolerance to several different stresses. Taken together, all the results summarized here give interesting perspectives for improving NUE and tolerance to multiple abiotic stresses in many crops by targeting both GS enzymes.

**Table 1:** Transgenic approaches manipulating amino acid metabolism and transport to improve nitrogen use efficiency in crops.

Genes	Gene source	Promoter	Target plant	Phenotype observed	References
Glutamine synthetase					
*GS1b*	*Medicago sativa*	*CaMV35S*	*Lotus japonicus*	Increase plant biomass and N uptake at the vegetative stage	Ortega et al., 2004
			*Nicotiana tabacum*	Increase leaf AA content in high N conditions but not in low N conditions; No effect on plant growth, photosynthesis and chlorophyll content	Fuentes et al., 2001
*GS1*	*Pisum sativum*	*CaMV35S*	*Nicotiana tabacum*	Increase plant biomass at the vegetative stage in high, moderate and low N conditions	Oliveira et al., 2002
*GS1.2*	*Populus simonii x Populus nigra*	*CaMV35S*	*Nicotiana tabacum*	Increase plant biomass, photosynthesis, AA content and cell wall biosynthesis	Lu et al., 2018b
*GS1-5*	*Glycine max*	*CaMV35S*	*Nicotiana tabacum*	Decrease plant growth and final pod number per plant	Seger et al., 2015
		*CaMV35S*	*Pisum sativum*	No effect on plant biomass and N uptake	Fei et al., 2006
		*LBC*_3_	*Pisum sativum*	No effect on plant biomass and N uptake	
		*rolD*	*Pisum sativum*	Increase plant biomass and N uptake in high and low N conditions	
*GLN1-3*	*Zea mays*	*CsVMV*	*Zea mays*	Increase grain yield and grain number without affecting the thousand kernel weight and grain N content	Martin et al., 2006
*GLN1-2*	*Sorghum bicolor*	*Ubq*	*Sorghum bicolor*	Increase total biomass and grain number of greenhouse- grown or field-grown plants in high N conditions but not in low N conditions	Urriola and Rathore, 2015
*GS1.1*	*Oryza sativa*	*CaMV35S*	*Oryza sativa*	Increase N uptake and N assimilation; Decrease plant growth, total biomass and grain yield in high, moderate and low N conditions	Cai et al., 2009;Bao et al., 2014
*GS1.2*	*Oryza sativa*	*CaMV35S*	*Oryza sativa*		
*glnA*	*Escherichia coli*	*CaMV35S*	*Oryza sativa*		
*GS1.1*	*Hordeum vulgare*	*Cisgenic expression*	*Hordeum vulgare*	Increase plant NUE and grain yield under both high and low N inputs either in ambient or elevated CO_2_ conditions; decrease grain protein content	Gao et al., 2019
*GS1.1, GS2*	*Oryza sativa*	*CaMV35S*	*Oryza sativa*	Increase N assimilation and plant biomass; increase tolerance to drought, salt and PEG-based osmotic stress *via* a better N remobilization towards proline biosnthesis	James et al., 2018
*GS1*	*Triticum aestivum*	*CaMV35S*	*Nicotiana tabacum*	Increase tolerance to drought	Yu et al., 2020
*GS2*	*Triticum aestivum*	*CaMV35S*	*Nicotiana tabacum*		
NADH-Glutamate:2-oxoglutarate aminotransferase					
*NADH-GOGAT*	*Medicago sativa*	*CaMV35S*	*Nicotiana tabacum*	Increase plant biomass and total C and N contents at the flowering stage	Chichkova et al., 2001
*OsGOGAT1*	*Oryza sativa*	*endogenous*	*Oryza sativa*	Increase grain weight	Yamaya et al., 2002
*OsGOGAT1*	*Oryza sativa*	*Activation tagging lines*	*Oryza sativa*	Increase NUpE in low N conditions; increase N content of grains; decrease grain yield per plant	Lee et al., 2020a
*OsGOGAT1, OsAMT1*	*Oryza sativa*	*Activation tagging lines*	*Oryza sativa*	Increase NUpE in low N conditions; increase N content of grains; maintain grain yield per plant	
*NADH-GOGAT*	*Triticum aestivum*	*Actin1*	*Zea mays*	Decrease shoot dry weight and kernel yield	Canas et al., 2020
Asparagine synthetase					
*ASN1*	*Pisum sativum*	*CaMV35S*	*Nicotiana tabacum*	Increase asparagine biosynthesis without affecting overall plant biomass	Brears et al., 1993
*ASN1*	*Oryza sativa*	*Ubi*	*Oryza sativa*	Increase N content of grains; no impact on grain yield	Lee et al., 2020b
*ASN-A*	*Escherichia coli*	*CaMV35S*	*Brassica napus*	Decrease seed yield and N seed content in high and low N conditions	Seiffert et al., 2004
		*35S/MAS*	*Lactuca sativa*	Increase plant biomass; no impact on final seed yield per plant	Giannino et al., 2007
		*PCpea*	*Lycopersicum solanum*	Increase NUpE with different ratios of NO_3_/NH_4_ present in the soil; no impact on plant biomass	Martinez-Andujar et al., 2013
NADH-Glutamate dehygrogenase					
*legdh1*	*Lycopersicum solanum*	*CaMV35S*	*Nicotiana tabacum*	No impact on plant biomass	Purnell et al., 2005
*GDHA*	*Nicotiana plumbaginifolia*	*CaMV35S*	*Nicotiana tabacum*	Decrease plant biomass; increase tolerance to salt stress	Terce-Laforgue et al., 2013, 2015
*GDHB*					
*GDHA,GDHB*					
NADPH-Glutamate dehydrogenase					
*gdhA*	*Aspergillus niger*	*CaMV35S*	*Oryza sativa*	Increase ammonia assimilation and plant biomass under high N conditions; Increased grain yield under field conditions	Abiko et al., 2010
*gdhA*	*Aspergillus nidulans*	*CaMV35S*	*Solanum tuberosum*	Increase tuber number, tuber dry weight and carbon and nitrogen content per tuber in both moderate and low N conditions	Egami et al., 2012
*GDH*	*Pleurotus cystidiosus*	*Ubi*	*Oryza sativa*	Increase N assimilation and thousand grain weight under moderate and low N field conditions	Zhou et al., 2014
*GDH*	*Trichurus*	*Ubi*	*Oryza sativa*	Increase N assimilation, thousand grain weight, grain number and seed protein content under high, moderate and low N field conditions	Du et al., 2019
*GDH*	*Magnaporthe grisea*	*Ubi*	*Oryza sativa*	No impact on plant growth; increase tolerance to dehydration	Zhou et al., 2015
*GDH*	*Sclerotinia sclerotiorum*	*Ubi*	*Oryza sativa*	No impact on plant growth and grain yield; decrease seedling growth	Du et al., 2014
*gdhA*	*Escherichia coli*	*CaMV35S*	*Nicotiana tabacum*	Increase N uptake, N assimilation and plant biomass under both controlled and field conditions	Ameziane et al., 2000
		*Ubi*	*Zea mays*	Increase grain biomass production in field conditions; Improve tolerance to drought stress	Lightfoot et al., 2007
*Glycine decarboxylase complex/ serine hydroxymethyl aminotransferase*					
*GDCH1*	*Arabidopsis thaliana*	*ST-LS1*	*Nicotiana tabacum*	Increase photosynthesis and plant biomass	Lopez-Calcagno et al., 2019
		*CaMV35S*	*Nicotiana tabacum*	Decrease plant growth and biomass	
*SHMT1*	*Oryza sativa*	*actin*	*Oryza sativa*	Increased photosynthesis and grain number per panicle	Wu et al., 2015
*Alanine:2-oxoglutarate aminotransferase*					
*ALAAT*	*Hordeum vulgare*	*btg26*	*Brassica napus*	Increase nitrate influx, NUpE, plant biomass and seed yield in greenhouse conditions and in the field under low N conditions	Good et al., 2007
		*Ant1*	*Oryza sativa*	Increase plant biomass, NUpE and final seed yield under high N conditions independently of soil N source (ammonia/nitrate)	Shrawat et al., 2008
		*Ant1*	*Saccharum officinarum*	Increase plant biomass and NUE in low N conditions	Snyman et al., 2015
		*Ant1*	*Triticum aestivum*	Increase plant biomass and grain yield in moderate N conditions but not in low N conditions	Pena et al., 2017
		*UBI4*	*Triticum aestivum*		
		*Ant1*	*Sorghum bicolor*	No impact on plant biomass	
		*UBI4*	*Sorghum bicolor*		
*ALAAT2*	*Cucumis sativa*	*Ant1*	*Oryza sativa*	Increase NUpE and grain yield in high and moderate N conditions	Sisharmini et al., 2019
*Amino acid permease*					
*AAP1*	*Vicia faba*	*LeB4*	*Vicia narbonensis*	Increase N uptake from roots and seed protein content	Rolletschek et al., 2005
			*Pisum sativum*		
*AAP1*	*Pisum sativum*	*AAP1*	*Pisum sativum*	Increase NUpE and NUE under both high and low N conditions	Perchlik and Tegeder, 2017
*AAP1*	*Oryza sativa*	*CaMV35S*	*Oryza sativa*	Increase tiller number and grain yield	Ji et al., 2020
*AAP3*	*Oryza sativa*	*CaMV35S*	*Oryza sativa*	Decrease tiller number and grain yield	Lu et al., 2018a
*AAP3 RNAi* *AAP3 CRISPR*	*Oryza sativa*	*NA*	*Oryza sativa*	Increase bud outgrowth, tiller number and grain yield	
*AAP5*	*Oryza sativa*	*CaMV35S*	*Oryza sativa*	Decrease tiller number and grain yield	Wang et al., 2019a
*AAP5 RNAi* *AAP5 CRISPR*	*Oryza sativa*	*NA*	*Oryza sativa*	Increase bud outgrowth, tiller number and grain yield	
*AAP6a*	*Glycine max*	*CaMV35S*	*Glycine max*	Increase source-to-sink AA transport and N content in seeds under both high and low N conditions	Liu et al., 2020
		*endogenous*	*Glycine max*		
*AAP6*	*Oryza sativa*	*CaMV35S*	*Oryza sativa*	Increase AA uptake from roots, AA transport and grain protein content at final harvest; maintain grain yield	Peng et al., 2014

Regarding GOGAT engineering, overexpression of an endogenous NADH-GOGAT (*OsGOGAT1*) in *Oryza sativa* increased NUpE in low N conditions but grain yield was decreased by up to 50% (Table 1). Interestingly, pyramiding *OsGOGAT1* with a rice gene encoding an ammonium transporter succeeded to rescue this low grain yield phenotype. Unfortunately, this pyramiding strategy was not successful for maize. While the constitutive expression of NADH-GOGAT from wheat decreased shoot dry weight and kernel yield, the introduction of an alternative pathway to boost the biosynthesis of 2-oxoglutarate and glutamine (the two substrates of GOGAT) did not recue the negative phenotype (Canas et al., 2020). Overall, the results were somewhat deceiving, and they appear to suggest that GOGAT activity has a low control coefficient on N assimilation.

### Asparagine Synthetase

Asparagine synthetase (ASN) can produce asparagine either by the condensation of ammonia and aspartate or by the transamination of glutamine and aspartate. In *Arabidopsis*, ASN is encoded by three genes: *ASN1*, *ASN2*, and *ASN3* (Gaufichon et al., 2016). *ASN1* and *ASN2* are important for AA metabolism and transport (redistribution/remobilization) at the seed filling stage (Gaufichon et al., 2013, 2017). However, *ASN1* is preferentially induced by leaf senescence compared to *ASN2* and *ASN3* (Figure 1; Have et al., 2017). In *Arabidopsis*, constitutive overexpression of *ASN1* using a *CaMV35S* promoter enhanced N allocation to seeds by promoting the phloem source-to-sink remobilization of asparagine and significantly increased the thousand seed weight (Lam et al., 2003; Gaufichon et al., 2017).

Since plant ASNs play a minor role in primary N assimilation (compared to GS), an interesting strategy was the overexpression of a bacterial type-A isoform from *E. coli* (ASN-A), which can only use ammonia as an amide donor compared to plant ASNs. This led to an increase in biomass of *Lactuca sativa* and NUpE of *Lycopersicum solanum* but brought about opposite effects in transformed *Brassica napus* in both high and low N conditions (Table 1). However, *Brassica napus* has a highly dynamic apoplastic NH_4_ pool, which may explain a higher sensibility to an accumulation of ammonia triggered by ASN-A activity (Nielsen and Schjoerring, 1998). A second strategy has consisted of boosting N reallocation to seeds by overexpressing plant ASNs. Indeed, an increase in total N, protein, and AA content of seeds without affecting grain yield per plant was observed when *OsASN1* was overexpressed in *Oryza sativa* (Lee et al., 2020b). Although plant ASNs have been shown already to represent interesting targets to modulate seed quality without affecting plant biomass, their potential for NUE improvement under low N conditions must still be addressed.

### Glutamate Dehydrogenase

Glutamate dehydrogenase (GDH) is involved in ammonia assimilation and production by catalyzing the reversible amination of 2-oxoglutarate to glutamate. Plant GDH is located in the cytosol and mitochondria as a multiprotein complex comprising three NAD(H)-dependent subunits (α, β, and γ) encoded by three genes in *Arabidopsis* (*GDH1*, *GDH2*, and *GDH3*). A fourth gene *GDH4* encoding a putative NADP(H)-dependent GDH has been identified in *Arabidopsis* but its exact catalytic activity and function are both still not clear (Igarashi et al., 2009). Although NADH-GDH can incorporate ammonia into 2-oxoglutarate to form glutamate in response to high levels of ammonia under stress conditions (Skopelitis et al., 2006), the major catalytic activity of NADH-GDH in plant cells is restricted to glutamate deamination and thus ammonia production (Masclaux-Daubresse et al., 2006). However, NADH-GDH-mediated glutamate deamination in roots significantly contributes to AA catabolism and TCA cycle activity during dark-induced senescence and hypoxia recovery (Miyashita and Good, 2008; Fontaine et al., 2012; Diab and Limami, 2016). Without surprise, biotechnological engineering of higher plants by overexpressing plant GDH isoforms did not increase either plant biomass or NUpE (Table 1). In tobacco, a better tolerance to salt stress was observed, but plant biomass remained lower when compared to control lines in normal conditions. Consequently, some research groups focused their work on the overexpression of either bacterial or fungal NADPH-GDH (Table 1). Indeed, *Aspergillus niger* NADPH-GDH α-subunit (*gdhA*) exhibited a higher maximal velocity and affinity for ammonia when compared to plant NADH-GDH and even cytosolic GS1 (Abiko et al., 2010). Except for GDH from two fungi (*Sclerotinia sclerotiorum* and *Magnaporthe grisea*), this strategy gave strikingly results in *Solanum tuberosum*, *Oryza sativa*, *Nicotiana tabacum*, and *Zea mays*. All overexpression lines showed higher N assimilation when compared to control lines coupled to positive effects on either plant biomass and/or grain yield under field and/or low N conditions. Interestingly, fungal and bacterial GDH have a higher potential for the improvement of NUE in crops compared to plant GS1. Indeed, NAD(P)H-GDH activity produces glutamate, a major AA, which is transported and used in many transamination reactions, by using 2-oxoglutarate, an organic acid efficiently produced by the tricarboxylic acid (TCA) cycle, as a carbon skeleton to fix ammonia. Conversely, GS activity produces glutamine, a transported AA requiring GOGAT enzyme activity to be further converted to glutamate. Therefore, GOGAT activity may become limiting in GS1-overexpressing lines while in GDH-overexpressing lines N assimilation becomes directly connected to TCA cycle activity and mitochondrial respiration. Considering *Oryza sativa*, these interesting results with GDH-overexpressing lines may also question a potential toxic accumulation of ammonia in some plant GS1-overexpressing lines, since a strong bacterial/fungal GDH activity could also stimulate ammonia import. An issue that still needs to be addressed is the phenotypical differences observed when overexpressing different fungal GDHs in *Oryza sativa*. Indeed, these phenotypical differences cannot be explained by different kinetic parameters since recombinant GDH from *Aspergillus niger*, *Trichuris*, and *Sclerotinia sclerotiorum* exhibited similar Km values for NH_4_ and 2-oxoglutarate (Abiko et al., 2010; Du et al., 2014, 2019).

## Amino Acid Biosynthesis and Degradation

### Photorespiratory N-Related Enzymes

Under current atmospheric conditions (415 μl.L^−1^), ribulose-1,5-bisphosphate carboxylase/oxygenase (Rubisco) oxygenase activity produces 2-phosphoglycolate, which is an inhibitor of Calvin-Benson cycle enzymes (Flugel et al., 2017). The photorespiratory cycle metabolizes this toxic 2-phosphoglycolate to make useful 3-phosphoglycerate, a metabolite hub of C metabolism (Hodges et al., 2016; Timm and Hagemann, 2020). This important pathway takes place in all photosynthetic cells and contributes to the production and degradation of glycine and serine through the successive action of the following enzymes: glutamate:glyoxylate aminotransferase (GGAT), glycine decarboxylase complex (GDC), serine hydroxymethyl aminotransferase (SHMT), and serine:glyoxylate aminotransferase (SGAT). In *Arabidopsis*, it has been shown that *GGT1*, *SHMT1*, and *SGAT1* are the photorespiratory genes (Somerville and Ogren, 1980; Voll et al., 2006; Dellero et al., 2015, 2016). GDC is a multiprotein complex comprising four subunits which are also involved in other enzymatic complexes: the P-protein encoded by a single gene (*GDP1*), the L-protein encoded by two genes (*LPD1* and *LPD2*), the T-protein encoded by a single gene (*GDT1*), and the H-protein encoded by three genes (*GDCH1*, *GDCH2*, and *GDCH3*; Douce et al., 2001; Engel et al., 2007). Considering that the GDC step releases CO_2_ and ammonia and that photorespiration cannot be completely suppressed, researchers have improved plant productivity by introducing alternative pathways to optimize the refixation of photorespiratory CO_2_ and to minimize ammonia losses (Maurino, 2019).

The recent overexpression of specific subunits of the photorespiratory GDC complex intriguingly gave promising results that suggested GDC to be the rate-limiting step of the cycle (Timm and Hagemann, 2020; Figure 1, Table 1). Indeed, *Arabidopsis* lines overexpressing *LPD* or *GDCH* genes exhibited an increase in net CO_2_ assimilation rate and overall plant biomass by up to 20% at the vegetative stage compared to control lines (Timm et al., 2012, 2015). In tobacco, similar improvements were achieved in field conditions but only when GDCH1 overexpression was driven by a leaf-specific and light-regulated promoter (Lopez-Calcagno et al., 2019). Perhaps surprisingly, the constitutive overexpression of *OsSHMT1* in rice significantly increased photosynthetic efficiency and grain number per panicle by up to 25% (Wu et al., 2015), whereas the overexpression of either *SGAT1* or *GGAT1* in *Arabidopsis* did not increase plant biomass (Igarashi et al., 2006; Modde et al., 2017).

Given these results, it is tempting to think that the phenotype of plants overexpressing subunits of the GDC complex or SHMT1 may be due to a modified flux through the photorespiratory cycle thus reducing the accumulation of toxic photorespiratory intermediates rather than an altered glycine/serine metabolism. Indeed, lower amounts of 2-phosphoglycolate would improve Calvin-Benson cycle activity, RuBisCO net CO_2_ fixation, and ultimately plant growth and N management. That said, the overexpression of *PGLP1* in *Arabidopsis* did not significantly increase plant biomass and photosynthetic performances under current atmospheric conditions (21% O_2_; Flugel et al., 2017). However, these transgenic lines showed a higher photosynthetic rate when compared to control lines without changing their water use efficiency under drought stress (Timm et al., 2019). As yet, we do not know whether overexpressing GDC subunits or SHMT1 confers an advantage under drought stress or low N inputs. This perspective may be promising for improving plant NUE with respect to climate change conditions.

### Alanine:2-Oxoglutarate Aminotransferase

Plant alanine:2-oxoglutarate aminotransferase (ALAAT) catalyzes the reversible transamination of glutamate and pyruvate to produce 2-oxoglutarate and alanine in the cytosol and mitochondria of various cell types. In *Arabidopsis*, mitochondrial ALAAT is encoded by two genes: *ALAAT1*, which is strongly expressed in vascular tissues of shoots and roots compared to *ALAAT2*, which is weakly expressed in vascular tissues of shoots (Ricoult et al., 2006; Miyashita et al., 2007). Interestingly, *ALAAT1* is induced in roots when plants are facing hypoxia stress such as waterlogging, where it plays an essential role in storing C and N skeletons and optimizing ATP production (Diab and Limami, 2016).

A successful strategy for crop improvement consisted in the root specific expression of a barley *HvALAAT1* cDNA (Table 1). This led to an increase in NUpE, plant biomass, and seed yield in *Brassica napus*, *Oryza sativa*, *Sacharum officinarum*, and *Triticum aestivum*, although these results were sometimes only observed in high N conditions or low N conditions depending on the crop tested. In addition, neither root-specific nor constitutive expression of *HvALAAT1* using an ubiquitin promoter succeeded to increase plant biomass in *Sorghum bicolor*, and thus suggesting that ALAAT activity was not rate-limiting for NUE in *Sorghum bicolor*. Recent results have confirmed a link between a higher ALAAT activity in roots and improved N-related phenotypes since the expression of ALAAT2 from *Cucumis sativus*, ALAAT1 and ALAAT2 from *Mus musculus*, and ALAAT from *Pyrococcus furiosus* in either *Arabidopsis* or *Oryza sativa* successfully increased plant growth in high or low N conditions (McAllister and Good, 2015; Sisharmini et al., 2019). How ALAAT activity leads to improved agronomical performance of plants has not been clearly elucidated (McAllister et al., 2012). However, based on some of the phenotypes observed in overexpressing lines, ALAAT could contribute to plant fitness in several ways. First, ALAAT may stimulate glycolysis and thereafter TCA cycle activity in roots by actively consuming pyruvate. Second, ALAAT may boost N uptake and assimilation in roots by actively recycling glutamate to 2-oxoglutarate for GOGAT activity. Finally, overproduction of alanine through ALAAT activity may efficiently activate alanine transport between plant organs thereby increasing the sink demand for N.

### Amino Acid Catabolism During Leaf Senescence

**Figure 2:**
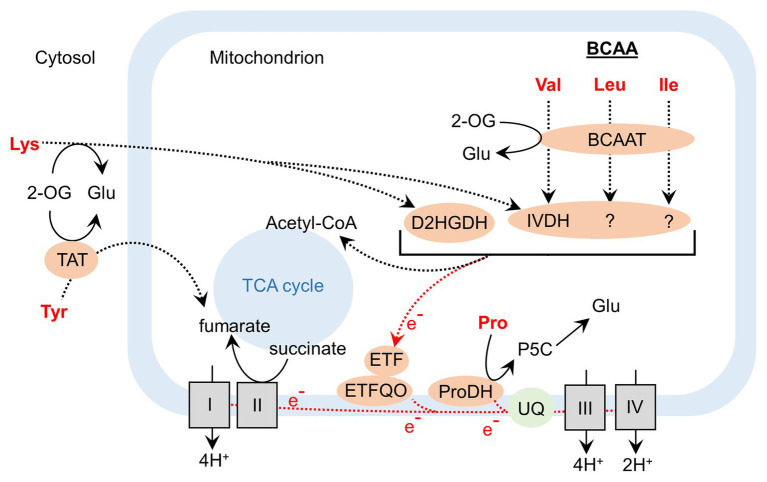
Contribution of amino acid catabolism to the activity of the tricarboxylic acid cycle and to the mitochondrial electron transfer chain during dark-induced senescence in *Arabidopsis*. BCAAT, branched-chain amino acid aminotransferase; D2HGDH, D-2-hydroxyglutarate dehydrogenase; ETF, electron-transfer flavoprotein; ETFQO, electron-transfer flavoprotein:ubiquinone oxidoreductase; IVDH, isovaleryl-CoA dehydrogenase ProDH, proline dehydrogenase; P5C, 1-pyrroline-5-carboxylic acid; TAT, tyrosine aminotransferase; TCA, tricarboxylic acid; UQ, ubiquinone; 2-OG, 2-oxoglutarate.

The regulation of the balance of AA and protein biosynthesis and degradation in plant cells is of primary importance to support different plant growth stages (Hildebrandt et al., 2015). Specifically, when cells are facing senescence or adverse stresses, the activation of autophagy processes results in an intense degradation of stored proteins (Masclaux-Daubresse et al., 2017). This mechanism allows plants to remobilize C and N for the production of valuable compounds for stress tolerance/adaptation, for the production of energy, and for the establishment of sink tissues (Hildebrandt, 2018; Tegeder and Masclaux-Daubresse, 2018). Interestingly, developmental and dark-induced senescence (prolonged dark stress) share common pathways for the regulation of AA metabolism and this includes the activation of the mitochondrial catabolism of branched-chain amino acids (BCAAs; namely leucine, valine, and isoleucine; Chrobok et al., 2016; Law et al., 2018; Figure 1). This metabolic pathway is of primary importance since it recycles the N of each BCAA while redirecting their C skeleton to the TCA cycle in the form of acetyl-CoA. At the same time, this AA catabolism actively participates in mitochondrial energy production, by providing electrons to a flavoprotein of the ubiquinone complex of the mitochondrial electron transfer chain (METC; Figure 2; Araujo et al., 2010; Hildebrandt et al., 2015). Since ammonia reassimilation during senescence and AA transport both require ATP to function, BCAA catabolism represents a promising target to boost source-to-sink N remobilization in plants by remobilizing N from BCAAs while participating in mitochondrial ATP production.

Besides BCAAs, the catabolism of other AAs can also contribute to mitochondrial energy production during dark-induced senescence (Figure 2). The characterization of a D-2-hydroxyglutarate dehydrogenase (D2HGDH) *Arabidopsis* mutant *d2hgdh* coupled to ^13^C-lysine labeling experiments confirmed that lysine degradation *via* D2HGDH supplied electrons to the METC during dark-induced senescence (Araujo et al., 2010). Recently, the analysis of *Arabidopsis* tyrosine aminotransferase 1 mutants showed that Tyr degradation significantly contributed to TCA cycle activity during dark-induced senescence by supplying fumarate (Wang et al., 2019b). Proline dehydrogenase (ProDH), the first enzyme of mitochondrial degradation of proline, has also attracted attention. In *Arabidopsis*, ProDH1 can form part of a low-molecular-mass complex in the mitochondrial membrane and actively contribute to the METC by acting as an electron donor (Cabassa-Hourton et al., 2016). The analysis of a *prodh1prodh2* double mutant confirmed that the proline oxidation significantly contributed to the METC during dark-induced senescence (Launay et al., 2019). In oilseed rape, certain *BnProDH* homologous genes (*BnaA&CProDH1a*, *BnaA&CProDH1b*, *BnaAProDH2a*, and *BnaCProDH2a*) were induced by prolonged dark conditions (Dellero et al., 2020a). However, variations of proline content between sink and source-to-senescent leaves at the vegetative stage were mostly correlated to a reduction of proline biosynthesis rather than to an increase of proline oxidation (Dellero et al., 2020a,b). This recent finding has questioned the importance of proline catabolism with respect to crop improvement. In potato, the short-term inhibition of the activity of the 2-oxoglutarate dehydrogenase complex (involved in the production of succinate in the TCA cycle) with a phosphonate analog resulted in a severe inhibition of mitochondrial respiration (Araujo et al., 2008). Nevertheless, long-term downregulation of this complex by antisense inhibition in tomato slightly reduced plant biomass and flowering time without affecting final fruit size, thereby suggesting that METC activity required for fruit production can be supported by alternative substrates (Araujo et al., 2012). Since isoleucine, leucine, lysine, proline, valine, and tyrosine are used as alternative respiratory substrates for mitochondrial respiration in plants during dark-induced senescence, enzymes of their respective catabolic pathways represent interesting and promising targets to boost source-to-sink N remobilization in plants. Extending this work to other crops will definitely provide exciting results on the exact contribution of their catabolism on plant NUE and perhaps under low N conditions.

## Amino Acid Uptake and Transport

During vegetative growth and reproductive stages, AAs are transported from soil to roots (AA uptake) and between roots, shoots, and sink tissues through the vascular tissues of xylem and phloem. AA transport from roots to shoots is apoplasmically achieved by the xylem and xylem loading is expected to be a passive step since it is facilitated by the difference of water potential between roots and leaves (Rennie and Turgeon, 2009). AA transport from source leaves to sink tissues is symplasmically achieved through the phloem. However, phloem loading and unloading events and xylem-to-phloem loading can also follow an apoplasmic route, which require the activity of specific plasma membrane AA transporters (Tegeder and Masclaux-Daubresse, 2018). These transporters are involved in the cellular import of a broad range of AAs in co-transport with protons, and they belong to five multigene families: amino acid permeases (AAP; eight genes in *Arabidopsis*), cationic amino acid transporters (CAT; nine genes in *Arabidopsis*), lysine histidine transporters (LHT; ten genes in *Arabidopsis*), proline transporters (ProT; three genes in *Arabidopsis*), and “usually multiple amino acid move in and out transporters” (UMAMIT; 43 genes in *Arabidopsis*; Su et al., 2004; Lehmann et al., 2011; Tegeder and Ward, 2012; Dinkeloo et al., 2018).

Reverse genetic approaches based on the characterization of *Arabidopsis* mutants have succeeded to identify the function of many AA transporters in recent years (Figure 1). Concerning AA uptake, the disruption of *AAP1*, *AAP5*, *LHT1*, *LHT6*, or *ProT2* expression successfully decreased AA uptake capacities in the corresponding transgenic plants (Lee et al., 2007; Svennerstam et al., 2007, 2011; Lehmann et al., 2011; Perchlik et al., 2014). In addition, AAP1 can contribute to salt stress-induced proline uptake while LHT1 plays a role in AA import into leaf cells (Hirner et al., 2006; Wang et al., 2017). However, the importance of AAP1 for crop improvement can be questioned since potato *StAAP1* antisense lines showed no differences with respect to C and N contents in seeds and overall seed yield when compared to control lines (Koch et al., 2003). Concerning AA export from either roots or source leaves, AAP2 and AAP6 play a key role in xylem-to-phloem loading of AAs while the role of AAP3 seems to be easily compensated by other AA transporters (Figure 1; Okumoto et al., 2004; Hunt et al., 2010; Zhang et al., 2010). AAP8 and UMAMIT18 are involved in phloem loading of AA for leaf export (Figure 1; Ladwig et al., 2012; Santiago and Tegeder, 2016, 2017). Interestingly, deletion of *Arabidopsis AAP8* decreased AA exudation from leaves at both vegetative and reproductive stages and ultimately decreased seed yield by up to 40% compared to the control line. Concerning AA import into seeds, UMAMIT11 and UMAMIT14 are both responsible for phloem unloading of AAs to seeds while UMAMIT24, UMAMIT25, UMAMIT28, and UMAMIT29 are involved in seed loading of AAs (Figure 1; Muller et al., 2015; Besnard et al., 2016, 2018). Perhaps surprisingly, single deletion of UMAMIT11, UMAMIT14, UMAMIT28, or UMAMIT29 decreased seed size at final harvest. From a global point of view, many AAP and UMAMIT transporters mentioned here are transcriptionally upregulated during natural leaf senescence and represent interesting targets for crop improvement (Figure 1; Have et al., 2017).

Up until now, biotechnological engineering of AA transport has been essentially focused on AAPs (Table 1). A successful strategy has consisted in the overexpression of *AAP1* and *AAP6* in different tissues (phloem, embryo, seed, or all plant organs) to improve AA transport from source-to-sink tissues in *Vicia narbonensis*, *Pisum sativum*, *Oryza sativa*, and *Glycine max*. Consequently, these overexpressing plants exhibited higher N root uptake and/or AA transport capacities in both high and low N conditions, thereby resulting in higher N seed content and/or seed yield. From a global view, similar phenotypes were observed by using promoters differing in strength and conferring different tissue localizations. This confirmed the multifunctional role of AAP1 and AAP6 in plant N metabolism and highlighted them as valuable targets for crop improvement. However, this overexpression strategy was not successful for other AAPs, hence reinforcing the interest of exploring the roles of AAPs specifically in crops. Indeed, the constitutive overexpression of *OsAAP5* and *OsAAP3* in rice gave negative NUE phenotypes while either RNA interference or genome-editing of these genes using CRISPR technologies (Clustered Regularly Interspaced Short Palindromic Repeats) increased bud outgrowth and tiller number by up to 30% and grain yield by up to 50%. Perhaps such results reflect the fact that *Os*AAP3 and *Os*AAP5 can promote the uptake of lysine and arginine from roots and these AAs can inhibit specifically root growth and axillary bud outgrowth in rice through the negative regulation of cytokinin production. Similarly, *Arabidopsis* knock-out lines for *AAP2* showed a better NUpE and seed yield under both high and low N conditions. Nevertheless, interesting phenotypes obtained by downregulating AA transport seems to be restricted to AAP only. In fact, the downregulation of *OsLHT1* in rice through either knock-out or CRISPR technology inhibited AA uptake from roots and their translocation to leaves while negatively impacting plant growth, and finally, seed yield by up to 50% (Wang et al., 2019c; Guo et al., 2020).

It can be seen above that the potential of AAP and UMAMIT genes for improving NUE in N-limiting conditions has not been fully investigated among crops while the recent genome-wide identification of *AAP* genes and their regulation during various stresses in many crops will definitely provide interesting targets for the future improvement of crop NUE (Ma et al., 2016; Wan et al., 2017; Qu et al., 2019; Duan et al., 2020; Zhou et al., 2020).

## Concluding Remarks and Perspectives

Over the two last decades, major advances have been achieved to improve NAE and NUE under high and low N conditions through the genetic manipulation of AA metabolism. Concerning NAE improvement, while plant GS1 has represented an interesting target, breakthrough results in low N conditions for many crops have been obtained mainly by expressing in bacterial ASN and fungal and bacterial GDH genes in plants. Indeed, plant GS1 activity is regulated at multiple levels and represents a rather high energetic cost; therefore, an uncontrolled increase of GS1 activity is prone to interfere with plant physiology and metabolic cell homeostasis. Probably bacterial ASN and fungal and bacterial GDH are insensitive to plant cell regulations and their *in vitro* affinity for ammonia appears to be much better when compared to GS1. Thus, an interesting strategy to maintain NAE while reducing field N inputs could consist in the overexpression of non-plant ASN and GDH isoforms. However, we do not know whether any beneficial effects in low N conditions would be conserved in the context of climate change, i.e., in combination with elevated CO_2_ and temperatures as well as prolonged periods of drought. Work performed using *Nicotiana tabacum*, *Hordeum vulgare*, and *Oryza sativa* clearly illustrated that plant GS1 remains an interesting target for this purpose (Table 1). Since a gene-pyramiding strategy for *OsGS1.1* and *OsGS2* in *Oryza sativa* gave attractive results, it might be interesting to boost ATP production while overexpressing plant GS1. For this purpose, targeting branched-chain AA catabolism may be a promising strategy. One may question the interest of boosting both AA biosynthesis and AA degradation. However, AA catabolism is often activated during stress-induced senescence and it has a high capacity for ATP production compared to the ATP required to reassimilate their N. The complete oxidation of a molecule of either BCAA, Lys, Pro, and Tyr releases around 29 ATP while the reassimilation of their N requires only 1 ATP (Hildebrandt et al., 2015). Therefore, this strategy could be ideal during senescence-inducing stress conditions by optimizing the redirection of carbon and nitrogen fluxes within plant cells. Specifically, the potential of IVDH, an enzyme that can participate in both lysine and valine catabolism, should be further evaluated within a gene pyramiding context.

Besides N assimilation and AA remobilization, AA transport represents an important rate-limiting step for NUE in many crops. This transport is of prime importance for NUE, as it affects N uptake, sink/source relationships, and ultimately seed N content. The assessment of the biotechnological potential of many AAPs in *Arabidopsis* has contributed to the development of genetic engineering approaches targeting AAPs in crops. However, results obtained with *Oryza sativa* confirmed that a strong AA transport activity can sometimes interfere with plant physiology (*via* cytokinin balance), and that this behavior is largely dependent on N metabolism topology in the tested crop. Of course, boosting AA uptake by overexpressing AAP1 and AAP6 in different tissues always resulted in a higher final seed N content in many different crops and even in low N conditions for the two leguminous species *Pisum sativum* and *Glycine max*. However, there was not always a positive impact on plant biomass or grain yield, perhaps due to limitations in sink size. In fact, successful engineering of sink/source relationships was mainly achieved through multi-target transformations improving both C/N remobilization and sink strength/size (Sonnewald and Fernie, 2018). It was recently shown that N reassimilation *via* the photorespiratory pathway increased plant net CO_2_ uptake (Busch et al., 2018). Therefore, overexpression of either AAP1/AAP6 in crops could be coupled with the overexpression of photorespiratory proteins at the interface between C and N metabolisms, such as GDCH and SHMT1.

The identification of new targets for genetic engineering represents a major challenge. Given that a systematic knock-out/overexpression approach for each potential target in different crops would represent a tedious and time-consuming strategy, the development of ^15^N-based fluxomic approaches using crops could help to identify novel, suitable metabolic steps to target for NUE improvement. Whole-plant ^15^N-based fluxomic approaches have been deployed already to analyze the impact of low N conditions on global NUpE and NUtE at the organ level and to identify the origin of N allocated to seeds (Have et al., 2017; Salon et al., 2017). Therefore, the further development of ^15^N-based fluxomics to explore AA metabolism in crops would be highly beneficial. For instance, the recent use of scalable metabolic flux modeling after ^15^N-glycine labeling showed that metabolic fluxes associated with *de novo* biosynthesis of proline and valine were strongly affected by leaf sink/source balances in *Brassica napus* (Dellero et al., 2020b). Future work should focus on developing more detailed and complete isotopic models of compartmented AA metabolism in crops under normal and low N conditions to identify new targets to improve NUE (and abiotic stress tolerance).

An important future challenge to consider regarding the improvement of crop NUE for sustainable agriculture is to propose non-GMO (Genetically Modified Organism) strategies. Indeed, genetically modified plants are not always publicly accepted especially in Europe due to a strong lobbying by environmental organizations. Forward genetic approaches based on natural genetic variation in crops have led to some interesting results for GS and AAPs. A QTL analysis of *Oryza sativa* identified *OsAAP6* as a positive regulator of grain protein content and AA uptake. Interestingly, the genetic diversity of grain protein content within *indica* cultivars was associated to two variations in the potential cis-regulatory elements of the *OsAAP6* 5'-untranslated region (Peng et al., 2014). Similarly, a genetic analysis in *Oryza sativa* identified an *indica* haplotype containing a 51 bp insertion in the promoter sequence of *AAP5* compared to *japonica* haplotypes. The *indica* haplotype had a lower *OsAAP5* transcript level and produced more tillers than *japonica* haplotypes (Wang et al., 2019a). Different genetic analysis in *Triticum aestivum* and *Zea mays* successfully identified GS-containing QTLs related to NUE parameters (N uptake and biomass; Fontaine et al., 2009; Li et al., 2015; Silva et al., 2018). In addition to these interesting perspectives, it is well-known that monocultural farming practices can impoverish the genetic diversity of soil microbiota, which is an important and overlooked actor of plant N metabolism by directly connecting plants to their neighboring environment. A major beneficial effect of bacteria on plant N acquisition is the symbiotic relationship between *rhizobia* and leguminous plants (Dellagi et al., 2020). While this bacteria-plant symbiotic interaction essentially occurs within Fabaceae, some plant-growth-promoting rhizobacteria and arbuscular mycorrhizal fungi with a wide-range of specificity for crops may have an interesting potential for the improvement of plant N nutrition that should be further evaluated.

## Author Contributions

The author confirms being the sole contributor of this work and has approved it for publication.

### Conflict of Interest

The author declares that the research was conducted in the absence of any commercial or financial relationships that could be construed as a potential conflict of interest.

## References

[ref1] Abiko T. Wakayama M. Kawakami A. Obara M. Kisaka H. Miwa T.. (2010). Changes in nitrogen assimilation, metabolism, and growth in transgenic rice plants expressing a fungal NADP(H)-dependent glutamate dehydrogenase (gdhA). Planta 232, 299–311. 10.1007/s00425-010-1172-3, PMID: 20443025

[ref2] Ameziane R. Bernhard K. Lightfoot D. (2000). Expression of the bacterial gdhA gene encoding NADPH glutamate dehydrogenase in tobacco affects plant growth and development. Plant Soil 221, 47–57. 10.1023/A:1004794000267

[ref3] Araujo W. L. Ishizaki K. Nunes-Nesi A. Larson T. R. Tohge T. Krahnert I.. (2010). Identification of the 2-hydroxyglutarate and isovaleryl-CoA dehydrogenases as alternative electron donors linking lysine catabolism to the electron transport chain of Arabidopsis mitochondria. Plant Cell 22, 1549–1563. 10.1105/tpc.110.075630, PMID: 20501910 PMC2899879

[ref4] Araujo W. L. Nunes-Nesi A. Nikoloski Z. Sweetlove L. J. Fernie A. R. (2012). Metabolic control and regulation of the tricarboxylic acid cycle in photosynthetic and heterotrophic plant tissues. Plant Cell Environ. 35, 1–21. 10.1111/j.1365-3040.2011.02332.x, PMID: 21477125

[ref5] Araujo W. L. Nunes-Nesi A. Trenkamp S. Bunik V. I. Fernie A. R. (2008). Inhibition of 2-oxoglutarate dehydrogenase in potato tuber suggests the enzyme is limiting for respiration and confirms its importance in nitrogen assimilation. Plant Physiol. 148, 1782–1796. 10.1104/pp.108.126219, PMID: 18842826 PMC2593666

[ref6] Avice J. C. Etienne P. (2014). Leaf senescence and nitrogen remobilization efficiency in oilseed rape (*Brassica napus* L.). J. Exp. Bot. 65, 3813–3824. 10.1093/jxb/eru177, PMID: 24790115

[ref7] Avila-Ospina L. Marmagne A. Talbotec J. Krupinska K. Masclaux-Daubresse C. (2015). The identification of new cytosolic glutamine synthetase and asparagine synthetase genes in barley (*Hordeum vulgare* L.), and their expression during leaf senescence. J. Exp. Bot. 66, 2013–2026. 10.1093/jxb/erv003, PMID: 25697791 PMC4378633

[ref8] Bao A. Zhao Z. Ding G. Shi L. Xu F. Cai H. (2014). Accumulated expression level of cytosolic glutamine synthetase 1 gene (OsGS1;1 or OsGS1;2) alter plant development and the carbon-nitrogen metabolic status in rice. PLoS One 9:e95581. 10.1371/journal.pone.0095581, PMID: 24743556 PMC3990726

[ref9] Behera S. N. Sharma M. Aneja V. P. Balasubramanian R. (2013). Ammonia in the atmosphere: a review on emission sources, atmospheric chemistry and deposition on terrestrial bodies. Environ. Sci. Pollut. Res. Int. 20, 8092–8131. 10.1007/s11356-013-2051-9, PMID: 23982822

[ref10] Besnard J. Pratelli R. Zhao C. Sonawala U. Collakova E. Pilot G.. (2016). UMAMIT14 is an amino acid exporter involved in phloem unloading in Arabidopsis roots. J. Exp. Bot. 67, 6385–6397. 10.1093/jxb/erw412, PMID: 27856708 PMC5181585

[ref11] Besnard J. Zhao C. Avice J. C. Vitha S. Hyodo A. Pilot G.. (2018). Arabidopsis UMAMIT24 and 25 are amino acid exporters involved in seed loading. J. Exp. Bot. 69, 5221–5232. 10.1093/jxb/ery302, PMID: 30312461 PMC6184519

[ref12] Bouchet A. S. Laperche A. Bissuel-Belaygue C. Baron C. Morice J. Rousseau-Gueutin M.. (2016). Genetic basis of nitrogen use efficiency and yield stability across environments in winter rapeseed. BMC Genet. 17:131. 10.1186/s12863-016-0432-z, PMID: 27628849 PMC5024496

[ref13] Brears T. Liu C. Knight T. J. Coruzzi G. M. (1993). Ectopic overexpression of asparagine synthetase in transgenic tobacco. Plant Physiol. 103, 1285–1290. 10.1104/pp.103.4.1285, PMID: 12232020 PMC159117

[ref14] Busch F. A. Sage R. F. Farquhar G. D. (2018). Plants increase CO_2_ uptake by assimilating nitrogen via the photorespiratory pathway. Nat. Plants 4, 46–54. 10.1038/s41477-017-0065-x, PMID: 29229957

[ref15] Cabassa-Hourton C. Schertl P. Bordenave-Jacquemin M. Saadallah K. Guivarc'h A. Lebreton S.. (2016). Proteomic and functional analysis of proline dehydrogenase 1 link proline catabolism to mitochondrial electron transport in *Arabidopsis thaliana*. Biochem. J. 473, 2623–2634. 10.1042/BCJ20160314, PMID: 27303048

[ref16] Cai H. Zhou Y. Xiao J. Li X. Zhang Q. Lian X. (2009). Overexpressed glutamine synthetase gene modifies nitrogen metabolism and abiotic stress responses in rice. Plant Cell Rep. 28, 527–537. 10.1007/s00299-008-0665-z, PMID: 19123004

[ref17] Canas R. A. Yesbergenova-Cuny Z. Belanger L. Rouster J. Brule L. Gilard F.. (2020). NADH-GOGAT overexpression does not improve maize (*Zea mays* L.) performance even when pyramiding with NAD-IDH, GDH and GS. Plants 9:130. 10.3390/plants9020130, PMID: 31973049 PMC7076717

[ref18] Chichkova S. Arellano J. Vance C. P. Hernández G. (2001). Transgenic tobacco plants that overexpress alfalfa NADH-glutamate synthase have higher carbon and nitrogen content. J. Exp. Bot. 52, 2079–2087. 10.1093/jexbot/52.364.2079 11604446

[ref19] Chrobok D. Law S. R. Brouwer B. Linden P. Ziolkowska A. Liebsch D.. (2016). Dissecting the metabolic role of mitochondria during developmental leaf senescence. Plant Physiol. 172, 2132–2153. 10.1104/pp.16.01463, PMID: 27744300 PMC5129728

[ref20] Dellagi A. Quillere I. Hirel B. (2020). Beneficial soil-borne bacteria and fungi: a promising way to improve plant nitrogen acquisition. J. Exp. Bot. 71, 4469–4479. 10.1093/jxb/eraa112, PMID: 32157312 PMC7475097

[ref21] Dellero Y. Clouet V. Marnet N. Pellizzaro A. Dechaumet S. Niogret M. F.. (2020a). Leaf status and environmental signals jointly regulate proline metabolism in winter oilseed rape. J. Exp. Bot. 71, 2098–2111. 10.1093/jxb/erz538, PMID: 31807778 PMC7242077

[ref22] Dellero Y. Heuillet M. Marnet N. Bellvert F. Millard P. Bouchereau A. (2020b). Sink/source balance of leaves influences amino acid pools and their associated metabolic fluxes in winter oilseed rape (*Brassica napus* L.). Meta 10:150. 10.3390/metabo10040150, PMID: 32295054 PMC7240945

[ref23] Dellero Y. Jossier M. Schmitz J. Maurino V. G. Hodges M. (2016). Photorespiratory glycolate-glyoxylate metabolism. J. Exp. Bot. 67, 3041–3052. 10.1093/jxb/erw090, PMID: 26994478

[ref24] Dellero Y. Lamothe-Sibold M. Jossier M. Hodges M. (2015). *Arabidopsis thaliana* ggt1 photorespiratory mutants maintain leaf carbon/nitrogen balance by reducing RuBisCO content and plant growth. Plant J. 83, 1005–1018. 10.1111/tpj.12945, PMID: 26216646

[ref25] Diab H. Limami A. M. (2016). Reconfiguration of N metabolism upon hypoxia stress and recovery: roles of alanine aminotransferase (AlaAT) and glutamate dehydrogenase (GDH). Plants 5:25. 10.3390/plants5020025, PMID: 27258319 PMC4931405

[ref26] Dinkeloo K. Boyd S. Pilot G. (2018). Update on amino acid transporter functions and on possible amino acid sensing mechanisms in plants. Semin. Cell Dev. Biol. 74, 105–113. 10.1016/j.semcdb.2017.07.010, PMID: 28705659

[ref27] Douce R. Bourguignon J. Neuburger M. Rebeille F. (2001). The glycine decarboxylase system: a fascinating complex. Trends Plant Sci. 6, 167–176. 10.1016/s1360-1385(01)01892-1, PMID: 11286922

[ref28] Du C. Q. Lin J. Z. Dong L. A. Liu C. Tang D. Y. Yan L.. (2019). Overexpression of an NADP(H)-dependent glutamate dehydrogenase gene, TrGDH, from Trichurus improves nitrogen assimilation, growth status and grain weight per plant in rice. Breed. Sci. 69, 429–438. 10.1270/jsbbs.19014, PMID: 31598075 PMC6776155

[ref29] Du C. Lin J. Yang Y. Liu H. Li C. Zhou Y.. (2014). Molecular cloning, characterization and function analysis of a GDH gene from Sclerotinia sclerotiorum in rice. Mol. Biol. Rep. 41, 3683–3693. 10.1007/s11033-014-3233-3, PMID: 24557889

[ref30] Duan Y. Zhu X. Shen J. Xing H. Zou Z. Ma Y.. (2020). Genome-wide identification, characterization and expression analysis of the amino acid permease gene family in tea plants (*Camellia sinensis*). Genomics 112, 2866–2874. 10.1016/j.ygeno.2020.03.026, PMID: 32276039

[ref31] Egami T. Wakayama M. Aoki N. Sasaki H. Kisaka H. Miwa T. (2012). The effects of introduction of a fungal glutamate dehydrogenase gene (*gdhA*) on the photosynthetic rates, biomass, carbon and nitrogen contents in transgenic potato. Plant Biotechnol. 29, 57–64. 10.5511/plantbiotechnology.12.0127a

[ref32] Engel N. van den Daele K. Kolukisaoglu U. Morgenthal K. Weckwerth W. Parnik T.. (2007). Deletion of glycine decarboxylase in Arabidopsis is lethal under nonphotorespiratory conditions. Plant Physiol. 144, 1328–1335. 10.1104/pp.107.099317, PMID: 17496108 PMC1914133

[ref33] Fei H. Chaillou S. Hirel B. Polowick P. Mahon J. D. Vessey J. K. (2006). Effects of the overexpression of a soybean cytosolic glutamine synthetase gene (GS15) linked to organ-specific promoters on growth and nitrogen accumulation of pea plants supplied with ammonium. Plant Physiol. Biochem. 44, 543–550. 10.1016/j.plaphy.2006.09.007, PMID: 17067806

[ref34] Flugel F. Timm S. Arrivault S. Florian A. Stitt M. Fernie A. R.. (2017). The Photorespiratory metabolite 2-Phosphoglycolate regulates photosynthesis and starch accumulation in Arabidopsis. Plant Cell 29, 2537–2551. 10.1105/tpc.17.00256, PMID: 28947491 PMC5774572

[ref35] Fontaine J. X. Ravel C. Pageau K. Heumez E. Dubois F. Hirel B.. (2009). A quantitative genetic study for elucidating the contribution of glutamine synthetase, glutamate dehydrogenase and other nitrogen-related physiological traits to the agronomic performance of common wheat. Theor. Appl. Genet. 119, 645–662. 10.1007/s00122-009-1076-4, PMID: 19513687

[ref36] Fontaine J. X. Terce-Laforgue T. Armengaud P. Clement G. Renou J. P. Pelletier S.. (2012). Characterization of a NADH-dependent glutamate dehydrogenase mutant of Arabidopsis demonstrates the key role of this enzyme in root carbon and nitrogen metabolism. Plant Cell 24, 4044–4065. 10.1105/tpc.112.103689, PMID: 23054470 PMC3517235

[ref37] Fuentes S. I. Allen D. J. Ortiz-Lopez A. Hernandez G. (2001). Over-expression of cytosolic glutamine synthetase increases photosynthesis and growth at low nitrogen concentrations. J. Exp. Bot. 52, 1071–1081. 10.1093/jexbot/52.358.1071, PMID: 11432923

[ref38] Gao Y. de Bang T. C. Schjoerring J. K. (2019). Cisgenic overexpression of cytosolic glutamine synthetase improves nitrogen utilization efficiency in barley and prevents grain protein decline under elevated CO_2_. Plant Biotechnol. J. 17, 1209–1221. 10.1111/pbi.13046, PMID: 30525274 PMC6576097

[ref39] Gaufichon L. Marmagne A. Belcram K. Yoneyama T. Sakakibara Y. Hase T.. (2017). ASN1-encoded asparagine synthetase in floral organs contributes to nitrogen filling in Arabidopsis seeds. Plant J. 91, 371–393. 10.1111/tpj.13567, PMID: 28390103

[ref40] Gaufichon L. Masclaux-Daubresse C. Tcherkez G. Reisdorf-Cren M. Sakakibara Y. Hase T.. (2013). *Arabidopsis thaliana* ASN2 encoding asparagine synthetase is involved in the control of nitrogen assimilation and export during vegetative growth. Plant Cell Environ. 36, 328–342. 10.1111/j.1365-3040.2012.02576.x, PMID: 22789031

[ref41] Gaufichon L. Rothstein S. J. Suzuki A. (2016). Asparagine metabolic pathways in Arabidopsis. Plant Cell Physiol. 57, 675–689. 10.1093/pcp/pcv184, PMID: 26628609

[ref42] Giannino D. Nicolodi C. Testone G. Frugis G. Pace E. Santamaria P. (2007). The overexpression of asparagine synthetase A from *E. coli* affects the nitrogen status in leaves of lettuce (*Lactuca sativa* L.) and enhances vegetative growth. Euphytica 162, 11–22. 10.1007/s10681-007-9506-3

[ref43] Good A. G. Johnson S. J. De Pauw M. Carroll R. T. Savidov N. Vidmar J. (2007). Engineering nitrogen use efficiency with alanine aminotransferase. Can. J. Bot. 85, 252–262. 10.1139/B07-019

[ref44] Guo N. Hu J. Yan M. Qu H. Luo L. Tegeder M.. (2020). *Oryza sativa* lysine-Histidine-type transporter 1 functions in root uptake and root-to-shoot allocation of amino acids in rice. Plant J. 103, 395–411. 10.1111/tpj.14742, PMID: 32159895

[ref45] Han M. Okamoto M. Beatty P. H. Rothstein S. J. Good A. G. (2015). The genetics of nitrogen use efficiency in crop plants. Annu. Rev. Genet. 49, 269–289. 10.1146/annurev-genet-112414-055037, PMID: 26421509

[ref46] Have M. Marmagne A. Chardon F. Masclaux-Daubresse C. (2017). Nitrogen remobilization during leaf senescence: lessons from Arabidopsis to crops. J. Exp. Bot. 68, 2513–2529. 10.1093/jxb/erw365, PMID: 27707774

[ref47] Hildebrandt T. M. (2018). Synthesis versus degradation: directions of amino acid metabolism during Arabidopsis abiotic stress response. Plant Mol. Biol. 98, 121–135. 10.1007/s11103-018-0767-0, PMID: 30143990

[ref48] Hildebrandt T. M. Nunes Nesi A. Araujo W. L. Braun H. P. (2015). Amino acid catabolism in plants. Mol. Plant 8, 1563–1579. 10.1016/j.molp.2015.09.005, PMID: 26384576

[ref49] Hirner A. Ladwig F. Stransky H. Okumoto S. Keinath M. Harms A.. (2006). Arabidopsis LHT1 is a high-affinity transporter for cellular amino acid uptake in both root epidermis and leaf mesophyll. Plant Cell 18, 1931–1946. 10.1105/tpc.106.041012, PMID: 16816136 PMC1533986

[ref50] Hodges M. Dellero Y. Keech O. Betti M. Raghavendra A. S. Sage R.. (2016). Perspectives for a better understanding of the metabolic integration of photorespiration within a complex plant primary metabolism network. J. Exp. Bot. 67, 3015–3026. 10.1093/jxb/erw145, PMID: 27053720

[ref51] Hunt E. Gattolin S. Newbury H. J. Bale J. S. Tseng H. M. Barrett D. A.. (2010). A mutation in amino acid permease AAP6 reduces the amino acid content of the Arabidopsis sieve elements but leaves aphid herbivores unaffected. J. Exp. Bot. 61, 55–64. 10.1093/jxb/erp274, PMID: 19755569 PMC2791111

[ref52] Igarashi D. Izumi Y. Dokiya Y. Totsuka K. Fukusaki E. Ohsumi C. (2009). Reproductive organs regulate leaf nitrogen metabolism mediated by cytokinin signal. Planta 229, 633–644. 10.1007/s00425-008-0858-2, PMID: 19048287

[ref53] Igarashi D. Tsuchida H. Miyao M. Ohsumi C. (2006). Glutamate:glyoxylate aminotransferase modulates amino acid content during photorespiration. Plant Physiol. 142, 901–910. 10.1104/pp.106.085514, PMID: 16950862 PMC1630728

[ref54] James D. Borphukan B. Fartyal D. Ram B. Singh J. Manna M.. (2018). Concurrent overexpression of OsGS1;1 and OsGS2 genes in transgenic Rice (*Oryza sativa* L.): impact on tolerance to abiotic stresses. Front. Plant Sci. 9:786. 10.3389/fpls.2018.00786, PMID: 29977247 PMC6021690

[ref55] Ji Y. Huang W. Wu B. Fang Z. Wang X. (2020). The amino acid transporter OsAAP1 mediates growth and grain yield by regulating neutral amino acids uptake and reallocation in *Oryza sativa*. J. Exp. Bot. 71, 4763–4777. 10.1093/jxb/eraa256, PMID: 32485736 PMC7410190

[ref56] Kant S. Bi Y. M. Rothstein S. J. (2011). Understanding plant response to nitrogen limitation for the improvement of crop nitrogen use efficiency. J. Exp. Bot. 62, 1499–1509. 10.1093/jxb/erq297, PMID: 20926552

[ref57] Koch W. Kwart M. Laubner M. Heineke D. Stransky H. Frommer W. B.. (2003). Reduced amino acid content in transgenic potato tubers due to antisense inhibition of the leaf Hþ/amino acid symporter StAAP1. Plant J. 33, 211–220. 10.1046/j.1365-313x.2003.01618.x, PMID: 12535336

[ref58] Ladwig F. Stahl M. Ludewig U. Hirner A. A. Hammes U. Z. Stadler R.. (2012). Siliques are Red1 from Arabidopsis acts as a bidirectional amino acid transporter that is crucial for the amino acid homeostasis of siliques. Plant Physiol. 158, 1643–1655. 10.1104/pp.111.192583, PMID: 22312005 PMC3320175

[ref59] Lam H. M. Wong P. Chan H. K. Yam K. M. Chen L. Chow C. M.. (2003). Overexpression of the ASN1 gene enhances nitrogen status in seeds of Arabidopsis. Plant Physiol. 132, 926–935. 10.1104/pp.103.020123, PMID: 12805621 PMC167031

[ref60] Launay A. Cabassa-Hourton C. Eubel H. Maldiney R. Guivarc’h A. Crilat E.. (2019). Proline oxidation fuels mitochondrial respiration during dark-induced leaf senescence in *Arabidopsis thaliana*. J. Exp. Bot. 70, 6203–6214. 10.1093/jxb/erz351, PMID: 31504781 PMC6859731

[ref61] Law S. R. Chrobok D. Juvany M. Delhomme N. Linden P. Brouwer B.. (2018). Darkened leaves use different metabolic strategies for senescence and survival. Plant Physiol. 177, 132–150. 10.1104/pp.18.00062, PMID: 29523713 PMC5933110

[ref62] Lee Y. H. Foster J. Chen J. Voll L. M. Weber A. P. Tegeder M. (2007). AAP1 transports uncharged amino acids into roots of Arabidopsis. Plant J. 50, 305–319. 10.1111/j.1365-313X.2007.03045.x, PMID: 17419840

[ref63] Lee S. Marmagne A. Park J. Fabien C. Yim Y. Kim S. J.. (2020a). Concurrent activation of OsAMT1;2 and OsGOGAT1 in rice leads to enhanced nitrogen use efficiency under nitrogen limitation. Plant J. 103, 7–20. 10.1111/tpj.14794, PMID: 32369636 PMC7383903

[ref64] Lee S. Park J. Lee J. Shin D. Marmagne A. Lim P. O.. (2020b). OsASN1 overexpression in Rice increases grain protein content and yield under nitrogen-limiting conditions. Plant Cell Physiol. 61, 1309–1320. 10.1093/pcp/pcaa060, PMID: 32384162 PMC7377344

[ref65] Lehmann S. Gumy C. Blatter E. Boeffel S. Fricke W. Rentsch D. (2011). In planta function of compatible solute transporters of the AtProT family. J. Exp. Bot. 62, 787–796. 10.1093/jxb/erq320, PMID: 20959625 PMC3003823

[ref66] Li H. M. Liang H. Li Z. Tang Z. X. Fu S. L. Geng Y. Y.. (2015). Dynamic QTL analysis of protein content and glutamine synthetase activity in recombinant inbred wheat lines. Genet. Mol. Res. 14, 8706–8715. 10.4238/2015.July.31.19, PMID: 26345802

[ref67] Lightfoot D. A. Mungur R. Ameziane R. Nolte S. Long L. Bernhard K. (2007). Improved drought tolerance of transgenic *Zea mays* plants that express the glutamate dehydrogenase gene (gdhA) of *E. coli*. Euphytica 156, 103–116. 10.1007/s10681-007-9357-y

[ref68] Liu S. Wang D. Mei Y. Xia T. Xu W. Zhang Y.. (2020). Overexpression of GmAAP6a enhances tolerance to low nitrogen and improves seed nitrogen status by optimizing amino acid partitioning in soybean. Plant Biotechnol. J. 18, 1749–1762. 10.1111/pbi.13338, PMID: 31945255 PMC7336375

[ref69] Lopez-Calcagno P. E. Fisk S. Brown K. L. Bull S. E. South P. F. Raines C. A. (2019). Overexpressing the H-protein of the glycine cleavage system increases biomass yield in glasshouse and field-grown transgenic tobacco plants. Plant Biotechnol. J. 17, 141–151. 10.1111/pbi.12953, PMID: 29851213 PMC6330538

[ref70] Lothier J. Gaufichon L. Sormani R. Lemaitre T. Azzopardi M. Morin H.. (2011). The cytosolic glutamine synthetase GLN1;2 plays a role in the control of plant growth and ammonium homeostasis in Arabidopsis rosettes when nitrate supply is not limiting. J. Exp. Bot. 62, 1375–1390. 10.1093/jxb/erq299, PMID: 20959627

[ref71] Lu T. Liu L. Wei M. Liu Y. Qu Z. Yang C.. (2018b). The effect of poplar PsnGS1.2 overexpression on growth, secondary cell wall, and fiber characteristics in tobacco. Front. Plant Sci. 9:9. 10.3389/fpls.2018.00009, PMID: 29403519 PMC5780347

[ref72] Lu K. Wu B. Wang J. Zhu W. Nie H. Qian J.. (2018a). Blocking amino acid transporter OsAAP3 improves grain yield by promoting outgrowth buds and increasing tiller number in rice. Plant Biotechnol. J. 16, 1710–1722. 10.1111/pbi.12907, PMID: 29479779 PMC6131477

[ref73] Ma H. Cao X. Shi S. Li S. Gao J. Ma Y.. (2016). Genome-wide survey and expression analysis of the amino acid transporter superfamily in potato (*Solanum tuberosum* L.). Plant Physiol. Biochem. 107, 164–177. 10.1016/j.plaphy.2016.06.007, PMID: 27289266

[ref74] Martin A. Lee J. Kichey T. Gerentes D. Zivy M. Tatout C.. (2006). Two cytosolic glutamine synthetase isoforms of maize are specifically involved in the control of grain production. Plant Cell 18, 3252–3274. 10.1105/tpc.106.042689, PMID: 17138698 PMC1693956

[ref75] Martinez-Andujar C. Ghanem M. E. Albacete A. Perez-Alfocea F. (2013). Response to nitrate/ammonium nutrition of tomato (*Solanum lycopersicum* L.) plants overexpressing a prokaryotic NH4(+)-dependent asparagine synthetase. J. Plant Physiol. 170, 676–687. 10.1016/j.jplph.2012.12.011, PMID: 23394787

[ref76] Masclaux-Daubresse C. Chen Q. Have M. (2017). Regulation of nutrient recycling via autophagy. Curr. Opin. Plant Biol. 39, 8–17. 10.1016/j.pbi.2017.05.001, PMID: 28528166

[ref77] Masclaux-Daubresse C. Daniel-Vedele F. Dechorgnat J. Chardon F. Gaufichon L. Suzuki A. (2010). Nitrogen uptake, assimilation and remobilization in plants: challenges for sustainable and productive agriculture. Ann. Bot. 105, 1141–1157. 10.1093/aob/mcq028, PMID: 20299346 PMC2887065

[ref78] Masclaux-Daubresse C. Reisdorf-Cren M. Pageau K. Lelandais M. Grandjean O. Kronenberger J.. (2006). Glutamine synthetase-glutamate synthase pathway and glutamate dehydrogenase play distinct roles in the sink-source nitrogen cycle in tobacco. Plant Physiol. 140, 444–456. 10.1104/pp.105.071910, PMID: 16407450 PMC1361315

[ref79] Maurino V. G. (2019). Using energy-efficient synthetic biochemical pathways to bypass photorespiration. Biochem. Soc. Trans. 47, 1805–1813. 10.1042/BST20190322, PMID: 31754693

[ref80] McAllister C. H. Beatty P. H. Good A. G. (2012). Engineering nitrogen use efficient crop plants: the current status. Plant Biotechnol. J. 10, 1011–1025. 10.1111/j.1467-7652.2012.00700.x, PMID: 22607381

[ref81] McAllister C. H. Good A. G. (2015). Alanine aminotransferase variants conferring diverse NUE phenotypes in *Arabidopsis thaliana*. PLoS One 10:e0121830. 10.1371/journal.pone.0121830, PMID: 25830496 PMC4382294

[ref82] Miyashita Y. Dolferus R. Ismond K. P. Good A. G. (2007). Alanine aminotransferase catalyses the breakdown of alanine after hypoxia in *Arabidopsis thaliana*. Plant J. 49, 1108–1121. 10.1111/j.1365-313X.2006.03023.x, PMID: 17319845

[ref83] Miyashita Y. Good A. G. (2008). NAD(H)-dependent glutamate dehydrogenase is essential for the survival of *Arabidopsis thaliana* during dark-induced carbon starvation. J. Exp. Bot. 59, 667–680. 10.1093/jxb/erm340, PMID: 18296429

[ref84] Modde K. Timm S. Florian A. Michl K. Fernie A. R. Bauwe H. (2017). High serine:glyoxylate aminotransferase activity lowers leaf daytime serine levels, inducing the phosphoserine pathway in Arabidopsis. J. Exp. Bot. 68, 643–656. 10.1093/jxb/erw467, PMID: 28011718 PMC5441925

[ref85] Moison M. Marmagne A. Dinant S. Soulay F. Azzopardi M. Lothier J.. (2018). Three cytosolic glutamine synthetase isoforms localized in different-order veins act together for N remobilization and seed filling in Arabidopsis. J. Exp. Bot. 69, 4379–4393. 10.1093/jxb/ery217, PMID: 29873769 PMC6093384

[ref86] Muller B. Fastner A. Karmann J. Mansch V. Hoffmann T. Schwab W.. (2015). Amino acid export in developing Arabidopsis seeds depends on UmamiT facilitators. Curr. Biol. 25, 3126–3131. 10.1016/j.cub.2015.10.038, PMID: 26628011

[ref87] Nielsen K. Schjoerring J. K. (1998). Regulation of Apoplastic NH4 ⴙ concentration in leaves of oilseed rape. Plant Physiol. 118, 1361–1368. 10.1104/pp.118.4.1361, PMID: 9847110 PMC34752

[ref88] Okumoto S. Koch W. Tegeder M. Fischer W. N. Biehl A. Leister D.. (2004). Root phloem-specific expression of the plasma membrane amino acid proton co-transporter AAP3. J. Exp. Bot. 55, 2155–2168. 10.1093/jxb/erh233, PMID: 15361541

[ref89] Oliveira I. C. Brears T. Knight T. J. Clark A. Coruzzi G. M. (2002). Overexpression of cytosolic glutamine synthetase. Relation to nitrogen, light, and photorespiration. Plant Physiol. 129, 1170–1180. 10.1104/pp.020013, PMID: 12114571 PMC166511

[ref90] Ortega J. L. Temple S. J. Bagga S. Ghoshroy S. Sengupta-Gopalan C. (2004). Biochemical and molecular characterization of transgenic Lotus japonicus plants constitutively over-expressing a cytosolic glutamine synthetase gene. Planta 219, 807–818. 10.1007/s00425-004-1292-8, PMID: 15197594 PMC3881563

[ref91] Pena P. A. Quach T. Sato S. Ge Z. Nersesian N. Dweikat I. M.. (2017). Molecular and phenotypic characterization of transgenic wheat and sorghum events expressing the barley alanine aminotransferase. Planta 246, 1097–1107. 10.1007/s00425-017-2753-1, PMID: 28801748

[ref92] Peng B. Kong H. Li Y. Wang L. Zhong M. Sun L.. (2014). OsAAP6 functions as an important regulator of grain protein content and nutritional quality in rice. Nat. Commun. 5:4847. 10.1038/ncomms5847, PMID: 25209128 PMC4175581

[ref93] Perchlik M. Foster J. Tegeder M. (2014). Different and overlapping functions of Arabidopsis LHT6 and AAP1 transporters in root amino acid uptake. J. Exp. Bot. 65, 5193–5204. 10.1093/jxb/eru278, PMID: 25005136 PMC4157705

[ref94] Perchlik M. Tegeder M. (2017). Improving plant nitrogen use efficiency through alteration of amino acid transport processes. Plant Physiol. 175, 235–247. 10.1104/pp.17.00608, PMID: 28733388 PMC5580756

[ref95] Purnell M. P. Skopelitis D. S. Roubelakis-Angelakis K. A. Botella J. R. (2005). Modulation of higher-plant NAD(H)-dependent glutamate dehydrogenase activity in transgenic tobacco via alteration of beta subunit levels. Planta 222, 167–180. 10.1007/s00425-005-1510-z, PMID: 15803323

[ref96] Qu Y. Ling L. Wang D. Zhang T. Guo C. (2019). Genome-wide identification and expression analysis of the AAAP family in *Medicago truncatula*. Genetica 147, 185–196. 10.1007/s10709-019-00062-6, PMID: 30905050

[ref97] Rennie E. A. Turgeon R. (2009). A comprehensive picture of phloem loading strategies. Proc. Natl. Acad. Sci. U. S. A. 106, 14162–14167. 10.1073/pnas.0902279106, PMID: 19666555 PMC2729037

[ref98] Ricoult C. Echeverria L. O. Cliquet J. B. Limami A. M. (2006). Characterization of alanine aminotransferase (AlaAT) multigene family and hypoxic response in young seedlings of the model legume *Medicago truncatula*. J. Exp. Bot. 57, 3079–3089. 10.1093/jxb/erl069, PMID: 16899523

[ref99] Rolletschek H. Hosein F. Miranda M. Heim U. Gotz K. P. Schlereth A.. (2005). Ectopic expression of an amino acid transporter (VfAAP1) in seeds of Vicia narbonensis and pea increases storage proteins. Plant Physiol. 137, 1236–1249. 10.1104/pp.104.056523, PMID: 15793070 PMC1088317

[ref100] Salon C. Avice J. C. Colombie S. Dieuaide-Noubhani M. Gallardo K. Jeudy C.. (2017). Fluxomics links cellular functional analyses to whole-plant phenotyping. J. Exp. Bot. 68, 2083–2098. 10.1093/jxb/erx126, PMID: 28444347

[ref101] Santiago J. P. Tegeder M. (2016). Connecting source with sink: the role of Arabidopsis AAP8 in phloem loading of amino acids. Plant Physiol. 171, 508–521. 10.1104/pp.16.00244, PMID: 27016446 PMC4854717

[ref102] Santiago J. P. Tegeder M. (2017). Implications of nitrogen phloem loading for carbon metabolism and transport during Arabidopsis development. J. Integr. Plant Biol. 59, 409–421. 10.1111/jipb.12533, PMID: 28296149

[ref103] Seger M. Gebril S. Tabilona J. Peel A. Sengupta-Gopalan C. (2015). Impact of concurrent overexpression of cytosolic glutamine synthetase (GS1) and sucrose phosphate synthase (SPS) on growth and development in transgenic tobacco. Planta 241, 69–81. 10.1007/s00425-014-2165-4, PMID: 25213117

[ref104] Seiffert B. Zhou Z. Wallbraun M. Lohaus G. Mollers C. (2004). Expression of a bacterial asparagine synthetase gene in oilseed rape (*Brassica napus*) and its effect on traits related to nitrogen efficiency. Physiol. Plant. 121, 656–665. 10.1111/j.1399-3054.2004.00361.x

[ref105] Shrawat A. K. Carroll R. T. DePauw M. Taylor G. J. Good A. G. (2008). Genetic engineering of improved nitrogen use efficiency in rice by the tissue-specific expression of alanine aminotransferase. Plant Biotechnol. J. 6, 722–732. 10.1111/j.1467-7652.2008.00351.x, PMID: 18510577

[ref106] Silva I. T. Abbaraju H. K. R. Fallis L. P. Liu H. Lee M. Dhugga K. S. (2018). Biochemical and genetic analyses of N metabolism in maize testcross seedlings: 2. Roots. Theor. Appl. Genet. 131, 1191–1205. 10.1007/s00122-018-3071-0, PMID: 29541827 PMC5945762

[ref107] Sisharmini A. Apriana A. Khumaida N. Trijatmiko K. R. Purwoko B. S. (2019). Expression of a cucumber alanine aminotransferase2 gene improves nitrogen use efficiency in transgenic rice. J. Genet. Eng. Biotechnol. 17:9. 10.1186/s43141-019-0010-7, PMID: 31712914 PMC6848643

[ref108] Skopelitis D. S. Paranychianakis N. V. Paschalidis K. A. Pliakonis E. D. Delis I. D. Yakoumakis D. I.. (2006). Abiotic stress generates ROS that signal expression of anionic glutamate dehydrogenases to form glutamate for proline synthesis in tobacco and grapevine. Plant Cell 18, 2767–2781. 10.1105/tpc.105.038323, PMID: 17041150 PMC1626620

[ref109] Snyman S. J. Hajari E. Watt M. P. Lu Y. Kridl J. C. (2015). Improved nitrogen use efficiency in transgenic sugarcane: phenotypic assessment in a pot trial under low nitrogen conditions. Plant Cell Rep. 34, 667–669. 10.1007/s00299-015-1768-y, PMID: 25686580

[ref110] Somerville C. Ogren W. (1980). Photorespiration mutants of *Arabidopsis thaliana* deficient in serine-glyoxylate aminotransferase activity. Proc. Natl. Acad. Sci. U. S. A. 77, 2684–2687. 10.1073/pnas.77.5.2684, PMID: 16592821 PMC349467

[ref111] Sonnewald U. Fernie A. R. (2018). Next-generation strategies for understanding and influencing source-sink relations in crop plants. Curr. Opin. Plant Biol. 43, 63–70. 10.1016/j.pbi.2018.01.004, PMID: 29428477

[ref112] Su Y. H. Frommer W. B. Ludewig U. (2004). Molecular and functional characterization of a family of amino acid transporters from Arabidopsis. Plant Physiol. 136, 3104–3113. 10.1104/pp.104.045278, PMID: 15377779 PMC523371

[ref113] Suzuki A. Knaff D. B. (2005). Glutamate synthase: structural, mechanistic and regulatory properties, and role in the amino acid metabolism. Photosynth. Res. 83, 191–217. 10.1007/s11120-004-3478-0, PMID: 16143852

[ref114] Svennerstam H. Ganeteg U. Bellini C. Nasholm T. (2007). Comprehensive screening of Arabidopsis mutants suggests the lysine histidine transporter 1 to be involved in plant uptake of amino acids. Plant Physiol. 143, 1853–1860. 10.1104/pp.106.092205, PMID: 17293438 PMC1851813

[ref115] Svennerstam H. Jamtgard S. Ahmad I. Huss-Danell K. Nasholm T. Ganeteg U. (2011). Transporters in Arabidopsis roots mediating uptake of amino acids at naturally occurring concentrations. New Phytol. 191, 459–467. 10.1111/j.1469-8137.2011.03699.x, PMID: 21453345

[ref116] Tegeder M. Masclaux-Daubresse C. (2018). Source and sink mechanisms of nitrogen transport and use. New Phytol. 217, 35–53. 10.1111/nph.14876, PMID: 29120059

[ref117] Tegeder M. Ward J. M. (2012). Molecular evolution of plant AAP and LHT amino acid transporters. Front. Plant Sci. 3:21. 10.3389/fpls.2012.00021, PMID: 22645574 PMC3355764

[ref118] Terce-Laforgue T. Bedu M. Dargel-Grafin C. Dubois F. Gibon Y. Restivo F. M.. (2013). Resolving the role of plant glutamate dehydrogenase: II. Physiological characterization of plants overexpressing the two enzyme subunits individually or simultaneously. Plant Cell Physiol. 54, 1635–1647. 10.1093/pcp/pct108, PMID: 23893023

[ref119] Terce-Laforgue T. Clement G. Marchi L. Restivo F. M. Lea P. J. Hirel B. (2015). Resolving the role of plant NAD-glutamate dehydrogenase: III. Overexpressing individually or simultaneously the two enzyme subunits under salt stress induces changes in the leaf metabolic profile and increases plant biomass production. Plant Cell Physiol. 56, 1918–1929. 10.1093/pcp/pcv114, PMID: 26251210

[ref120] Thomsen H. C. Eriksson D. Moller I. S. Schjoerring J. K. (2014). Cytosolic glutamine synthetase: a target for improvement of crop nitrogen use efficiency? Trends Plant Sci. 19, 656–663. 10.1016/j.tplants.2014.06.002, PMID: 25017701

[ref121] Timm S. Florian A. Arrivault S. Stitt M. Fernie A. R. Bauwe H. (2012). Glycine decarboxylase controls photosynthesis and plant growth. FEBS Lett. 586, 3692–3697. 10.1016/j.febslet.2012.08.027, PMID: 22982108

[ref122] Timm S. Hagemann M. (2020). Photorespiration - how is it regulated and regulates overall plant metabolism? J. Exp. Bot. 71, 3955–3965. 10.1093/jxb/eraa183, PMID: 32274517

[ref123] Timm S. Wittmiss M. Gamlien S. Ewald R. Florian A. Frank M.. (2015). Mitochondrial Dihydrolipoyl dehydrogenase activity shapes photosynthesis and photorespiration of *Arabidopsis thaliana*. Plant Cell 27, 1968–1984. 10.1105/tpc.15.00105, PMID: 26116608 PMC4531348

[ref124] Timm S. Woitschach F. Heise C. Hagemann M. Bauwe H. (2019). Faster removal of 2-phosphoglycolate through photorespiration improves abiotic stress tolerance of Arabidopsis. Plants 8:563. 10.3390/plants8120563, PMID: 31810232 PMC6963629

[ref125] Urriola J. Rathore K. S. (2015). Overexpression of a glutamine synthetase gene affects growth and development in sorghum. Transgenic Res. 24, 397–407. 10.1007/s11248-014-9852-6, PMID: 25417185

[ref126] Voll L. M. Jamai A. Renne P. Voll H. McClung C. R. Weber A. P. (2006). The photorespiratory Arabidopsis shm1 mutant is deficient in SHM1. Plant Physiol. 140, 59–66. 10.1104/pp.105.071399, PMID: 16339799 PMC1326031

[ref127] Wallsgrove R. Turner J. Hall N. Kendall A. Bright S. (1987). Barley mutants lacking chloroplast glutamine Synthetase—biochemical and genetic analysis. Plant Physiol. 83, 155–158. 10.1104/pp.83.1.155, PMID: 16665193 PMC1056315

[ref128] Wan Y. King R. Mitchell R. A. C. Hassani-Pak K. Hawkesford M. J. (2017). Spatiotemporal expression patterns of wheat amino acid transporters reveal their putative roles in nitrogen transport and responses to abiotic stress. Sci. Rep. 7:5461. 10.1038/s41598-017-04473-3, PMID: 28710348 PMC5511167

[ref129] Wang T. Chen Y. Zhang M. Chen J. Liu J. Han H.. (2017). Arabidopsis AMINO ACID PERMEASE1 contributes to salt stress-induced Proline uptake from exogenous sources. Front. Plant Sci. 8:2182. 10.3389/fpls.2017.02182, PMID: 29312416 PMC5743684

[ref130] Wang M. Toda K. Block A. Maeda H. A. (2019b). TAT1 and TAT2 tyrosine aminotransferases have both distinct and shared functions in tyrosine metabolism and degradation in *Arabidopsis thaliana*. J. Biol. Chem. 294, 3563–3576. 10.1074/jbc.RA118.006539, PMID: 30630953 PMC6416433

[ref131] Wang J. Wu B. Lu K. Wei Q. Qian J. Chen Y.. (2019a). The amino acid Permease 5 (OsAAP5) regulates tiller number and grain yield in Rice. Plant Physiol. 180, 1031–1045. 10.1104/pp.19.00034, PMID: 30890663 PMC6548276

[ref132] Wang X. Yang G. Shi M. Hao D. Wei Q. Wang Z.. (2019c). Disruption of an amino acid transporter LHT1 leads to growth inhibition and low yields in rice. BMC Plant Biol. 19:268. 10.1186/s12870-019-1885-9, PMID: 31221084 PMC6584995

[ref133] Ward M. H. Jones R. R. Brender J. D. de Kok T. M. Weyer P. J. Nolan B. T.. (2018). Drinking water nitrate and human health: An updated review. Int. J. Environ. Res. Public Health 15:1557. 10.3390/ijerph15071557, PMID: 30041450 PMC6068531

[ref134] Wu J. Zhang Z. Zhang Q. Han X. Gu X. Lu T. (2015). The molecular cloning and clarification of a photorespiratory mutant, oscdm1, using enhancer trapping. Front. Genet. 6:226. 10.3389/fgene.2015.00226, PMID: 26191072 PMC4490251

[ref135] Xu G. Fan X. Miller A. J. (2012). Plant nitrogen assimilation and use efficiency. Annu. Rev. Plant Biol. 63, 153–182. 10.1146/annurev-arplant-042811-105532, PMID: 22224450

[ref136] Yamaya T. Obara M. Nakajima H. Sasaki S. Hayakawa T. Sato T. (2002). Genetic manipulation and quantitative-trait loci mapping for nitrogen recycling in rice. J. Exp. Bot. 53, 917–925. 10.1093/jexbot/53.370.917, PMID: 11912234

[ref137] Yoneyama T. Suzuki A. (2019). Exploration of nitrate-to-glutamate assimilation in non-photosynthetic roots of higher plants by studies of (15)N-tracing, enzymes involved, reductant supply, and nitrate signaling: a review and synthesis. Plant Physiol. Biochem. 136, 245–254. 10.1016/j.plaphy.2018.12.011, PMID: 30710774

[ref138] Yu H. Zhang Y. Zhang Z. Zhang J. Wei Y. Jia X.. (2020). Towards identification of molecular mechanism in which the overexpression of wheat cytosolic and plastid glutamine synthetases in tobacco enhanced drought tolerance. Plant Physiol. Biochem. 151, 608–620. 10.1016/j.plaphy.2020.04.013, PMID: 32335384

[ref139] Zhang X. Davidson E. A. Mauzerall D. L. Searchinger T. D. Dumas P. Shen Y. (2015). Managing nitrogen for sustainable development. Nature 528, 51–59. 10.1038/nature15743, PMID: 26595273

[ref140] Zhang L. Tan Q. Lee R. Trethewy A. Lee Y. H. Tegeder M. (2010). Altered xylem-phloem transfer of amino acids affects metabolism and leads to increased seed yield and oil content in Arabidopsis. Plant Cell 22, 3603–3620. 10.1105/tpc.110.073833, PMID: 21075769 PMC3015121

[ref141] Zhou Y. Liu H. Zhou X. Yan Y. Du C. Li Y. (2014). Over-expression of a fungal NADP(H)-dependent glutamate dehydrogenase PcGDH improves nitrogen assimilation and growth quality in rice. Mol. Breed. 34, 335–349. 10.1007/s11032-014-0037-z

[ref142] Zhou T. Yue C. P. Huang J. Y. Cui J. Q. Liu Y. Wang W. M.. (2020). Genome-wide identification of the amino acid permease genes and molecular characterization of their transcriptional responses to various nutrient stresses in allotetraploid rapeseed. BMC Plant Biol. 20:151. 10.1186/s12870-020-02367-7, PMID: 32268885 PMC7140331

[ref143] Zhou Y. Zhang C. Lin J. Yang Y. Peng Y. Tang D.. (2015). Over-expression of a glutamate dehydrogenase gene, MgGDH, from Magnaporthe grisea confers tolerance to dehydration stress in transgenic rice. Planta 241, 727–740. 10.1007/s00425-014-2214-z, PMID: 25486886

